# In Situ and Ex Situ Characterization of the Microstructure Formation in Ni-Cr-Si Alloys during Rapid Solidification—Toward Alloy Design for Laser Additive Manufacturing

**DOI:** 10.3390/ma13092192

**Published:** 2020-05-10

**Authors:** Xiaoshuang Li, Kai Zweiacker, Daniel Grolimund, Dario Ferreira Sanchez, Adriaan B. Spierings, Christian Leinenbach, Konrad Wegener

**Affiliations:** 1 Empa-Swiss Federal Laboratories for Materials Science and Technology, Überlandstrasse 129, CH-8600 Dübendorf, Switzerland; xiaoshuang.li@empa.ch (X.L.); kai.zweiacker@empa.ch (K.Z.); 2 Institute of Machine Tools and Manufacturing, ETH Zurich, Leonhardstrasse 21, CH-8092 Zurich, Switzerland; wegener@iwf.mavt.ethz.ch; 3 Swiss Light Source, Paul Scherrer Institut, CH-5232 Villigen PSI, Switzerland; daniel.grolimund@psi.ch (D.G.); Dario.Ferreira@psi.ch (D.F.S.); 4 Innovation Center for Additive Manufacturing Switzerland, Inspire AG, Lerchenfeldstrasse 3, CH-9014 St. Gallen, Switzerland; spierings@inspire.ethz.ch

**Keywords:** Ni-Cr-Si, rapid solidification, laser coating, anomalous eutectic

## Abstract

Laser beam-based deposition methods such as laser cladding or additive manufacturing of metals promises improved properties, performance, and reliability of the materials and therefore rely heavily on understanding the relationship between chemical composition, rapid solidification processing conditions, and resulting microstructural features. In this work, the phase formation of four Ni-Cr-Si alloys was studied as a function of cooling rate and chemical composition using a liquid droplet rapid solidification technique. Post mortem x-ray diffraction, scanning electron microscopy, and in situ synchrotron microbeam X-ray diffraction shows the present and evolution of the rapidly solidified microstructures. Furthermore, the obtained results were compared to standard laser deposition tests. In situ microbeam diffraction revealed that due to rapid cooling and an increasing amount of Cr and Si, metastable high-temperature silicides remain in the final microstructure. Due to more sluggish interface kinetics of intermetallic compounds than that of disorder solid solution, an anomalous eutectic structure becomes dominant over the regular lamellar microstructure at high cooling rates. The rapid solidification experiments produced a microstructure similar to the one generated in laser coating thus confirming that this rapid solidification test allows a rapid pre-screening of alloys suitable for laser beam-based processing techniques.

## 1. Introduction

Laser-aided manufacturing processes involving deposition of alloy powders on substrates have gained increasing interest in the recent past. Typical techniques include laser cladding or additive manufacturing (AM) technologies such as laser Direct Metal Deposition (DMD) or Selective Laser Melting (SLM). Laser-consolidated parts undergo complex thermal histories including rapid solidification of small material volumes with cooling rates in the range of 10^3^–10^4^ Ks^−1^ during cladding or DMD [1,2], and up to 10^6^–10^7^ K s^−1^ during SLM [3,4]. Large thermal gradients (G) and high solidification front velocities (V) during laser melting and subsequent rapid solidification process can result in non-equilibrium microstructures. This inherently includes non-equilibrium elemental partitioning or complete loss of elemental partitioning and the formation of metastable supersaturated phases [5,6,7,8]. The properties–performance relationship of laser-deposited coatings and laser-manufactured parts are determined by the complex interrelationships between the alloy composition, the processing parameters, and the resulting microstructure. However, the processing parameters in SLM are typically optimized to achieve dense and defect-free parts and are usually determined in a trial-and-error approach based on the Volume Energy Density (VED).

Recently, a lot of effort [9,10,11] has been made to develop optimized alloys with regards to the non-equilibrium conditions of laser processing. In this context, alloy pre-screening involves rapid solidification experiments to mimic the conditions during laser processing. This approach can be useful to overcome the costly and long route of producing new powders for newly developed alloys.

Kenel et al. [9] first proposed a laser melting setup in which small batch alloy samples of various sizes are molten and rapidly solidified. This method promised a fast technique to gain insights into the out-of-equilibrium microstructure. By combining experimental observations with heat transfer analysis based on finite element simulations, systemic rapid solidification studies with varying cooling rates can be realized. This approach gave unique insights into the non-equilibrium microstructural phase formation in Ti-Al and Ti-Al-(Nb, Mo) alloys [9,10]. The setup was then adapted for in situ synchrotron X-ray diffraction experiments to directly monitor the phase evolution during rapid melting and solidification as well as the subsequent cyclic laser heating and cooling. It is possible to elucidate the layer-by-layer fabrication of Ti-6Al-4V alloys [12]. Thus, the in situ melting setup is suitable to investigate new promising alloys for additive manufacturing.

Structural engineering parts in abrasive service conditions need to have wear and/or corrosion-resistant surface coating which remedies the deficient aspects of the substrate material. These alloys often contain hard transition metal silicides, which have high strength, excellent wear, corrosion, and oxidation resistance. Promising candidates for structural components or coatings have been outlined by Liu et al. [13].

For example, in the Ni-rich corner of the Ni-Cr-Si alloy system, several different silicides such as Ni3Si [14], Cr13Ni5Si2 [13] co-exist with the ductile (Ni) phase [13,15,16]. Various attempts to improve the performance of Ni-Cr-Si-based alloys for laser-deposited coatings were reported in [1,2,17,18,19,20,21]. The addition of Boron and Carbon into the Nickel-Chrome-Silicon system led to the development of the widely used hardfacing materials known as Colmonoy alloys [1]. These alloys have compositional ranges of chromium between 11 to 15 wt.%, Silicon, between 3 to 4 wt.%, Boron between 2 to 3 at.%, and Carbon between 0.48–0.76 wt.%. The resulting Ni-Cr-Si-B coating significantly improved the cavitation erosion-corrosion resistance alongside the mechanical properties of the steel due to the formation of borides and boro-carbides [22].

However, the formation of coarse carbides and cracking susceptibility are the biggest obstacles for viable use in commercial application of this series of hardfacing alloys. Alternatively, embedding abrasive particles, such as diamonds [18,23] or cemented carbide [17] into Ni-Cr-Si alloys produces an abrasive layer, where Ni-Cr-Si alloys serve as binding matrix material. Here, the survival of these abrasive particles is attributed to a short lifetime of melt pool during processing and the metallurgical bonding due to reactions with Cr and Si.

Recently, several authors successfully demonstrated that ternary Ni-Cr-Si [24] and quaternary Ni-Cr-Si-B alloys with various types of hard Cr silicides can be successfully deposited on Cu substrates by laser cladding. However, their studies are based on slight compositional modifications of commercial NiCrSiB alloys. It has not been studied if Ni-Cr-Si base alloys without B and C have the potential to form novel functional microstructures. In-depth microstructural investigations are necessary to investigate the performance, and determining microstructural features such as phase formation, phase constituents, phase-fraction, grain size, and grain morphology.

In this work, the resulting microstructures of several Ni-Cr-Si alloys after rapid solidification at different well-defined cooling rates between 1600 K s^−1^ and 11,000 K s^−1^ were characterized ex situ by scanning electron microscopy (SEM), X-ray diffraction (XRD), and in situ by synchrotron X-ray microbeam diffraction. In addition, a powder was prepared from one of the alloys and laser deposition tests were performed. The microstructure and hardness of the as-coated layer were compared with the results from the rapid solidification tests.

## 2. Materials and Methods

### 2.1. Alloy Selection

The Ni-Cr-Si-based alloys studied in this work were selected with regard to potential use in commercial laser deposition processes, thus the following criteria need to be considered:

- The alloys need to possess a small solidification range to reduce the susceptibility to solidification cracking [25,26]
- The alloys need to solidify with a (near-)eutectic microstructure, preferably with a fine lamellar rather than large, blocky phases to enhance the mechanical properties
- A sufficiently high amount of Cr for improved corrosion resistance
- A sufficiently high amount of Si for the formation of hard and wear-resistant silicides

**Figure 1:**
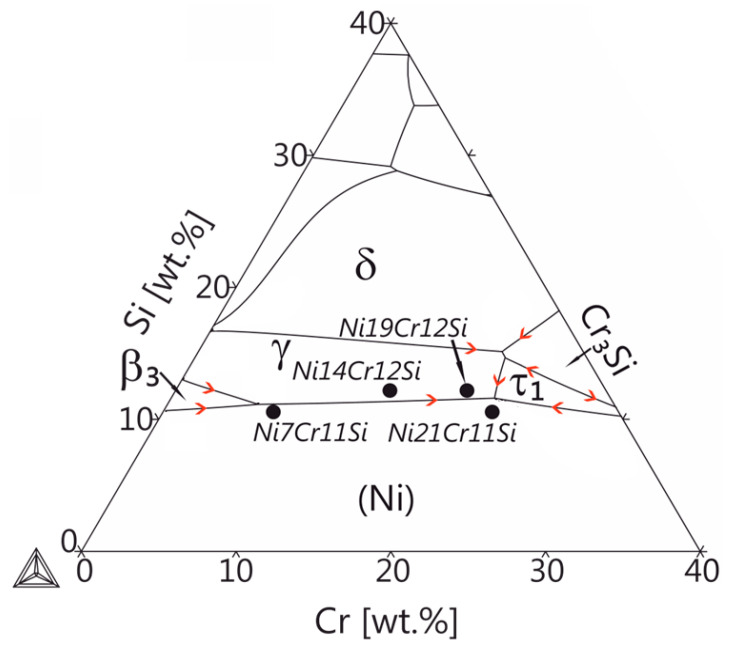
Liquidus projection of the ternary Ni-Cr-Si system at the Ni-rich corner. Thermodynamic database TCNi5 was used for calculations. Four alloys marked by black solid circles were selected for this study.

Figure 1 shows the liquidus projection of the Ni-rich corner of the Ni-Cr-Si ternary system, which was calculated using the Thermocalc software package in combination with the TCNi5 database. The selected alloys Ni7Cr11Si, Ni14Cr12Si, Ni19CrSi and Ni21Cr11Si are indicated by black dots. The phase fractions of the different phases upon solidification in the four different alloys were calculated using the Scheil-Gulliver model. All calculations were performed for a pressure P = 105 kPa.

### 2.2. Sample Preparation

Samples with a mass of about 2 g were prepared by arc melting. For this, Nickel slug (99.98%), chromium pieces (99.99%) supplied by Alfa-Aesar, Germany, and silicon chips (99.9999%) supplied by abcr GmbH, Germany, were used. The arc furnace was filled with 500 mbar Ar inert gas purified by an OXISORB cartridge (Messer). All alloys were molten twice and inverted to ensure homogeneity. This was repeated four times. The average weight loss due to melting and remelting was less than 0.2%. For the nominal composition, crystallographic parameters and the known equilibrium invariant reactions please refer to the supplementary materials Tables S1 and S2 in the supplementary file.

To perform solidification experiments at high cooling rates small amounts (3–230 mg) of the test alloys were prepared. Samples were cut from the master alloys and molten again in the arc furnace. This allowed producing semi-spherical samples of two different sizes, i.e., 0.5 and 2 mm in radius. The liquidus/solidus temperatures and latent heat of the alloys were measured by DSC (Netzsch Pegasus 404C, Netzsch-Gerätebau GmbH, Selb, Germany) using a heating/cooling rate of 10 K s^−1^. The results are listed in Table 1.

### 2.3. FE Simulations of Temperature Evolution during Rapid Cooling

The magnitude of the cooling rates, which proportionally relates to the solidification front velocity and the resulting microstructure, is inherently correlated with the sample dimensions. Thus, heat transfer processes involved in rapid solidification experiments were simulated by Finite Element Modeling (FEM) using Abaqus/CAE 6.13-2 (3DS Simulia) with a 160,000 hexahedral (DC3D8) element mesh. Details of the model are described in previous works [9,11]. The calculated liquidus/solidus temperatures and latent heat given in Table 1 were used.

**Figure 2:**
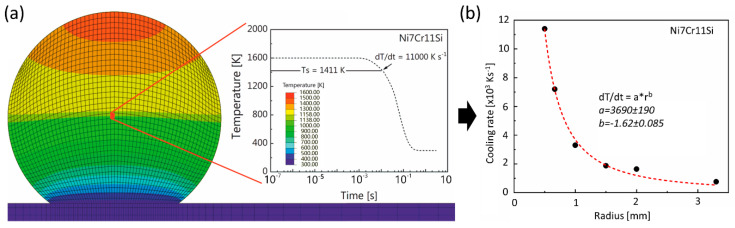
(**a**) Temperature distribution at the cross section of the sphere of 0.5 mm in radius and the temperature profile at the center of the sphere during rapid cooling, (**b**) Cooling rates for Ni7Cr11Si alloys of various sizes.

The emissivity of Ni-Cr binary alloys [27] was employed in the model. Other thermophysical properties, i.e., thermal conductivity [28], the temperature-dependent density [29], specific heat [30], were adapted from Ni-Cr alloys with similar amounts of solutes. In the model, the initial droplet temperature was set to 1600 K. The calculated cooling rates at the solidus and liquidus temperature are in the same order of magnitude, and the difference is relatively small. Therefore, the average cooling rates between liquidus and solidus temperature in the center of the sphere was considered in this work (cf. Figure 2a). Figure 2b summarizes the simulation effort and relates directly the cooling rate to the spherical dimensions of the liquid droplet. The subsequent microstructural analyses were also conducted at the position where the cooling rates were calculated. The cooling rates corresponding to the sample size of 0.5 and 2 mm radius are 11,000 K s^−1^ and 1600 K s^−1^, respectively.

### 2.4. In Situ Characterization of Rapid Phase Transformations by Synchrotron X-ray Micro-Diffraction

In situ synchrotron X-ray micro-beam-diffraction during rapid solidification was conducted at the microXAS beamline at the Swiss Light Source (SLS), Switzerland using the experimental setup for rapid solidification tests described in previous works [12,31]. Spheres of 0.5 mm radius corresponding to a cooling rate of 11,000 K s^−1^ were placed on a Cu tip for maximization of heat extraction and molten by two inclined diode lasers (λ = 980 nm) with an angle of 20° on two sides. Each laser can provide a maximum output power of 150 W. During heating and cooling, a beam with an energy of 17.3 keV and a spot size of 15 μm (horizontal) and 10 μm (vertical) continuously penetrated the specimens. The diffraction signals from the spheres during rapid heating and cooling were collected using an Eiger 4M single-photon counting detector (Dectris AG, Switzerland) with 2070 × 2167 pixels and a single-pixel size of 75 × 75 μm^2^. The detector operated at a frame rate of 333 Hz and 2.99 ms exposure time per frame. Recorded raw frames reveal scattered signals from amorphous and crystalline phases combined with features from beam optics, the specimen holder, the beam stop, defective pixels, and the module border that are removed by a background correction (seen in Figure S1 in the supplementary document). The duration of one heating and cooling cycle was 10 s. Shielding gas (Ar 4.8) was employed by a laminar flow nozzle in a radius of 15 mm above the specimen to avoid oxidation. Calibration of the experimental geometry was performed on standard α-Al_2_O_3_ powder. XRDUA v7.2.5.1 software was used for data analysis, including calibration and azimuthal integration.

### 2.5. Ex Situ Rapid Solidification Experiments and Post Mortem Characterization

Spherical samples with 0.5 and 2 mm were re-melted on a Cu substrate under similar conditions as the initial alloy production using the same setup as described in [9,11]. The electric arc was ignited directly above the pre-alloyed material and held for approximately 1s to fully melt the specimen. Subsequently, the arc was moved to maximum distance from the specimen to prevent overheating of the specimen and changing the microstructure due to different cooling rates.

To analyze the present phases polished specimens were characterized by X-ray diffraction (XRD) using a Bruker D8 DISCOVERY XRD (Bruker AXS GmbH, Karlsruhe, Germany) equipped with a LynxEye detector. The measurements were performed using Cu Kα radiation produced at an accelerating voltage of 40 kV and an electron current of 40 mA. A Ni-filter (0.012 mm) was used to absorb Cu Kβ emission and the step size was 0.02° (2θ). Pinhole snouts with diameters of 2 mm and 1 mm were used to reduce the beam size onto the small polished sample surfaces to minimize background noise. The microstructures were investigated using Scanning Electron Microscopy (SEM; FEI NanoSEM 230, now: ThermoFischer Scientific, Hillsboro, OR, USA) equipped with BSE and Energy Dispersive X-ray analysis (EDX).

### 2.6. Laser Deposition Experiments

To evaluate if rapid solidification experiments can mimic laser beam-based deposition processes, the Ni21Cr11Si alloy was selected for coating in a Sisma MySint 100 SLM machine. A master alloy of 10 g was produced in the way described in Section 2.1, and then it was manually crushed and ground in a tungsten carbide (WC) crucible. The powder was sieved with a stack of 20 and 40 μm sieves. Since laser coating trials on Ni21Cr11Si alloy focus on the obtained microstructure, regardless of defects, no spheroidization of powder took place. The laser processing parameters were as follows: P_l_ = 150 W; V_s_ = 1250 mm s^−1^; d_layer_ = 30 μm; d_hatch_ = 75 μm. A 316L stainless steel was used as base-plate material. Due to the small amount of powder, the recoating of powder was done manually for each layer after laser scanning. As a result, the laser coating process proceeded in an interrupted manner, which gave time for the preceding layer to cool down resulting in a higher thermal gradient than a continuous coating in the common SLM process.

## 3. Results and Discussion

### 3.1. Microstructure Characterization

#### 3.1.1. Phase Transformations during Rapid Cooling

To understand the rapid solidification process and the resulting microstructure, high energy synchrotron radiation was used to elucidate on the phase formation due to the inherent fast nature of the rapid solidification process.

**Figure 3:**
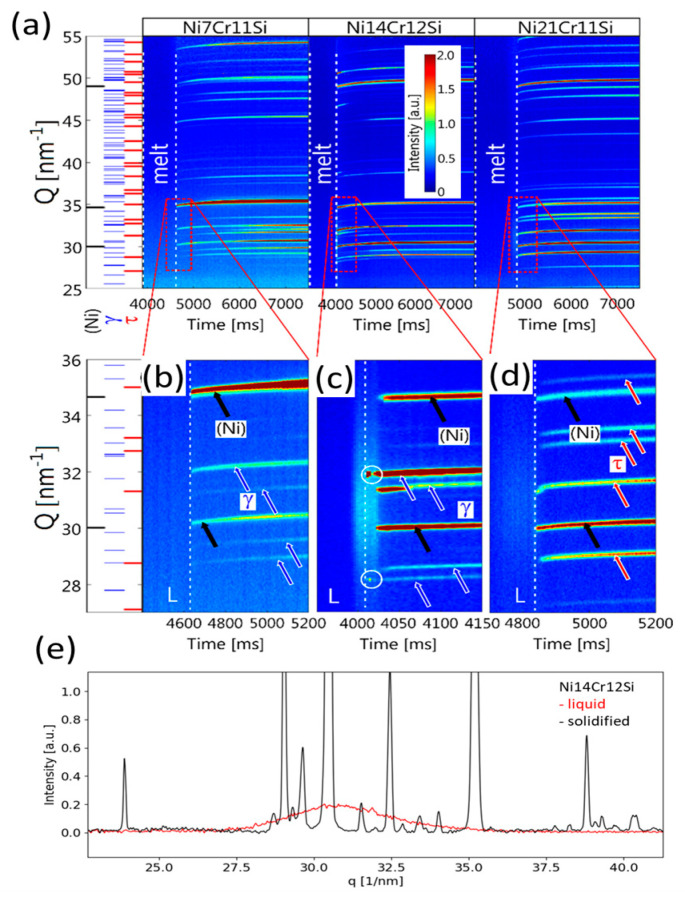
Synchrotron diffraction patterns were collected throughout the melting and the solidification cycle for three of the investigated alloys: Ni7Cr11Si, Ni14Cr12Si and Ni21Cr11Si. (**a**): 1D profile of diffraction pattern of Ni21Cr11Si melt; (**b**): Map of diffraction patterns; (**c**,**d**): Maps of diffraction patterns at the early stages of cooling; (**e**) diffraction pattern of liquid and solid Ni14Cr12Si.

In situ experiments were performed on spheres of 0.5 mm radius (i.e., 11,000 K s^−1^). Spheres with larger radii could not be penetrated with the X-ray beam. Figure 3 shows the evolution of the diffractograms during rapid solidification and subsequent cooling. In Figure 3a significant peak shifting can be observed throughout the experiments. The sudden appearance of peaks during cooling indicates the first formation of solid out of the liquid phase. The change in peak position indicates that during heat extraction the lattice parameters of all involved phases decrease by the thermal contraction and lead to decreased lattice spacing, or in reciprocal space to increased scattering vectors G_hkl_ of the diffraction peaks with Miller indices h, k, l. Furthermore, sudden changes in peak positions are indicative of the formation or decomposition of phases. In Figure 3a, no sharp diffraction peaks were observed in the melt zone between white dashed lines since no long-range order exists in the melt. The intensity of the broad amorphous peaks is only weakly observed, mainly due to the high extinction coefficient of the liquid alloy and the limited beam energy. However, the broad peaks are still distinguishable from the background in the 1D profile of Intensity vs. Q spacing pattern seen in Figure 3a for the Ni21Cr11Si alloy. In the investigated alloys, phase transformations occurred only during the first 500 ms of heat extraction after the solidification. Further decomposition of phases was not observed in the subsequent cooling of solid. Therefore, the graphs Figure 3b–d highlight the entire stages of solidification and the early onset of cooling. In both the Ni7Cr11Si and the Ni21Cr11Si alloy, peaks associated with two phases simultaneously appeared out of the melt which is an indication of an eutectic reaction. Two different eutectic reactions at high cooling rates are unveiled, i.e., L → (Ni) + γ-Ni_31_Si_12_ and L → (Ni) + τ-Cr_6_Ni_16_Si_7_. In comparison, peaks circled in Figure 3d reveals that a primary solid phase formation takes place over a time range prior to the eutectic reaction. Here γ-Ni_31_Si_12_ phase crystallized from the melt then followed by (Ni) + γ-Ni_31_Si_12_. The observed eutectic microstructure is attributed to the high cooling rate that leads to the formation of a pseudo-eutectic structure, i.e., a microstructure which has an eutectic morphology but not necessarily the eutectic composition. The solidification ranges in Ni7Cr11Si and Ni21Cr11Si alloys are smaller than that in Ni14Cr12Si alloy, so no liquid-solid phase-field was observed. For the rapid liquid to solid transformation, Figure 3e shows two temporal separated diffraction patterns of the Ni14Cr12Si at the same location. The red graph indicates that an amorphous phase is present in the diffraction volume, i.e., a liquid alloy. A diffraction pattern acquired shortly after the previous one was taken indicates that only crystalline material is present in the diffraction volume.

#### 3.1.2. Microstructure Formation during Rapid Heat Extraction from X-ray Analysis

**Figure 4:**
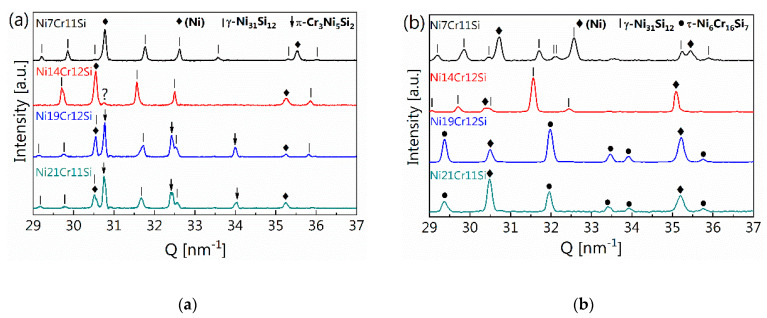
(**a**) Post mortem XRD patterns of the spheres with r = 2 mm (i.e., 1600 K s^−1^); (**b**) 1D profiles of synchrotron diffraction patterns of spheres with r = 0.5 mm (i.e., 11,000 K s^−1^). Compared with lab XRD device, the synchrotron gives a much better signal to background ratio, so the diffraction pattern collected at t = 0 s is plotted in (**b**). Q is the diffraction vector, i.e., momentum transfer in reciprocal space, which is a material characteristic parameter. The rhombus means the peaks for (Ni), solid line for γ-Ni31Si12 and dot for τ-Cr6Ni16Si7.

To understand the phase formation during rapid solidification processes in more detail, post mortem XRD measurements for all alloys were taken and are displayed in Figure 4 for two different heat extraction rates, i.e., Figure 4a at 1600 K s^−1^ and Figure 4b at 11,000 K s^−1^. The diffraction patterns displayed in Figure 4a were acquired using a parallel beam geometry probing planes with their diffraction vector k perpendicular to the solidification direction. This is in contrast to the diffraction patterns displayed in Figure 4b. Here, each diffraction pattern is derived from a 2D in situ synchrotron measurement which stem from azimuthally integrated 2D detector. The integrated diffraction patterns show better signal to noise ratio, possess better statistics for phase identification and quantitative analysis, and are less influenced by texture, large grain size, or small quantity. The post mortem XRD patterns of Ni7Cr11Si and Ni14Cr12Si rapidly solidified with a 1600 K s^−1^ thermal gradient in Figure 4a show that both alloys have similar phase components, i.e., (Ni) and γ-Ni_31_Si_12_. A shift of the distinct phase peaks toward higher Q values, which can be observed for the Ni7Cr11Si is presumably due to the solidification phase sequence and the lower Cr content in this sample, leading to the slightly smaller Ni unit cell dimensions. Comparing Ni19Cr12Si and Ni21Cr11Si, both alloys consist of three different phases, i.e., (Ni), γ-Ni_31_Si_12_ and π-Cr_3_Ni_5_Si_2_, respectively.

Moving on to the much higher cooling rate of about 11,000 K s^−1^, Figure 4b, the peaks visible in the diffraction pattern correspond to the τ-Cr_6_Ni_16_Si_7_ phase rather than the anticipated π-Cr_3_Ni_5_Si_2_ phase. Table 2 summarizes the observed and equilibrium anticipated phase constitutes of the investigated alloys for the various cooling rates.

#### 3.1.3. Microstructure Characterization Using SEM

**Figure 5:**
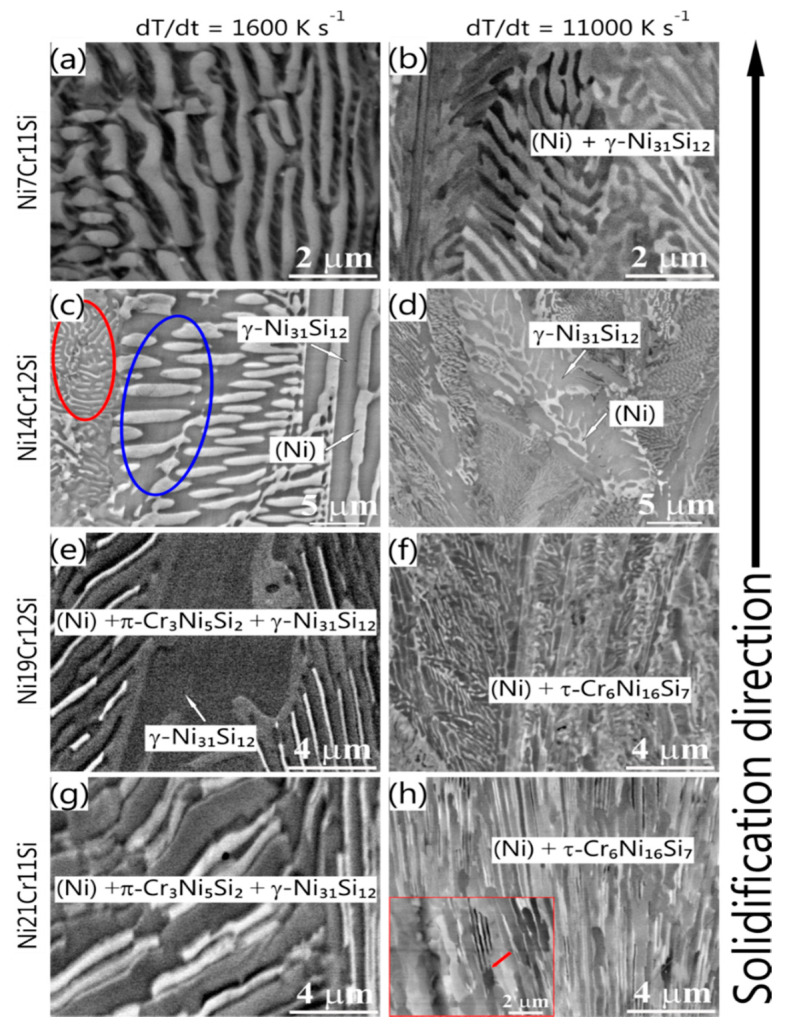
BSE micrographs of each rapidly solidified alloy, i.e., (**a**,**b**): Ni7Cr11Si; (**c**,**d**): Ni14Cr12Si; (**e**,**f**): Ni19Cr12Si; (**g**,**h**): Ni21Cr11Si. The microstructures on the left and right columns are obtained at a cooling rate of 1600 K s^−1^ and 11000 K s^−1^, respectively.

To confirm the findings from the X-ray analysis it is important to investigate microstructural feature size and morphology using electron beam-based techniques. According to the liquid projection line calculations (Figure 1) the primary (Ni) phase would be expected in the Ni7Cr11Si alloy. However, in Figure 5 a fully lamellar structure (Ni) + γ-Ni_31_Si_12_ is observed in all rapidly solidified spheres. Eutectic colonies with a feathery shape are elongated parallel to the heat extraction direction. Oscillation of lamellae with a discontinuous change in width was observed (Figure 5a,b) [32,33], which is assumed to be the result of an unstable solidification front due to a change in growth rate during the interface propagation [34]. Regular cooperative growth might have been interrupted due to the increase in interface solidification velocity leading up to a degenerated lamellar structure. Consequently, it is difficult to measure the spacing of discontinuous lamellae precisely, but it is still evident that the lamellar spacing, as well as the colony size, decreases with the increased cooling rate (see Figure 5). The growth of eutectic-like microstructure is determined by the experimental conditions, i.e., thermal gradient and chemical composition at the solid-liquid interface. The number of observed colonies increased with an increased cooling rate, indicating that the number of nuclei and nucleation rates increased. As nucleation is exponentially dependent on the driving force, i.e., the change of Gibbs free energy, a small change in undercooling results in significant change in nucleation rate. This implies that in current experimental condition undercooling increases with the increase of cooling rate.

The microstructure of Ni14Cr12Si spheres (Figure 5c,d) contains a mixture of large plate-like γ-Ni_31_Si_12_, fine lamellar eutectic (Ni) + γ-Ni_31_Si_12_ regions (highlighted by the red circle) and coarser, rod-like eutectic (Ni) + γ-Ni31Si12 regions (seen in blue circle), as confirmed by point EDX in combination with XRD measurements. The difference in crystal sizes in the different eutectic regions seems to be minimized at higher cooling rates and steeper temperature gradients.

In both the Ni19Cr12Si and Ni21Cr11Si alloy, a ternary eutectic microstructure (Ni) + π-Cr_3_Ni_5_Si_2_ + γ-Ni_31_Si_12_ is observed as shown in Figure 5e,g. It is interesting that an anomalous (Ni) + τ-Cr_6_Ni_16_Si_7_ eutectic-like structure is present in these two alloys at the highest cooling rate of 11000 K s^−1^. However, the morphology is very much different for the two different alloys, one is stripe as reported in [35] (Figure 5f) and the other is a mixture of fine lamellae and relatively coarse anomalous eutectic zones as seen in Figure 5h.

### 3.2. Microstructural Comparison of Commercially Used Ni21Cr11Si after Laser Deposition

**Figure 6:**
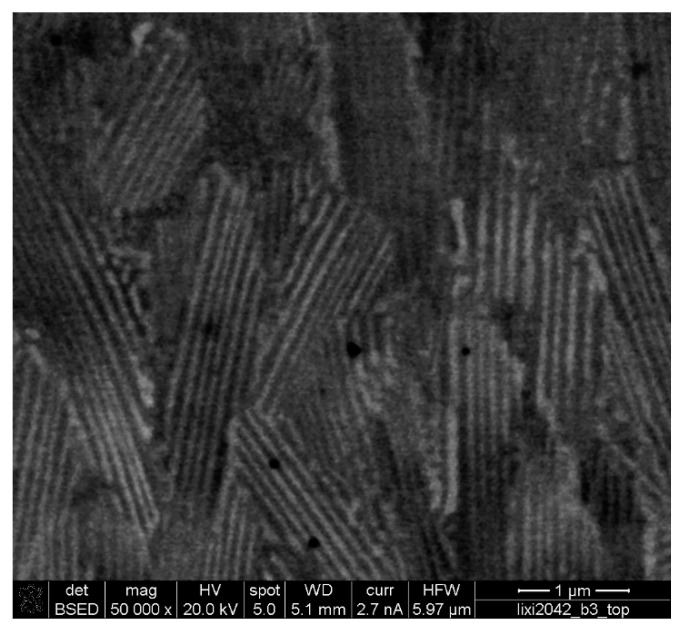
The microstructure of a laser coated Ni21Cr11Si showing the fine lamellar (Ni) + τ-Cr_6_Ni_16_Si_7_ grains.

Figure 6 shows the typical microstructure of Ni21Cr11Si after laser coating. The microstructure consists of fine lamellar (Ni) + τ-Cr_6_Ni_16_Si_7_ grains, which is consistent with the microstructure of the rapidly solidified sphere in Figure 5h. Therefore, it is reasonable to conclude that the microstructure formation has followed a very similar solidification pathway, thus confirming the validity of the present rapid solidification studies, which inherently can enable a better understanding of microstructural formation in the SLM process and acceleration of the corresponding alloy-screening phase specifically for laser-deposition applications.

## 4. Discussion

In the Ni7Cr11Si alloy, (Ni) formation is suppressed during rapid solidification. The grain growth of (Ni) ejects large amounts of Si to the melt, resulting in compositional modification at the solidification front that facilitates constitutional undercooling. It is likely that the large undercooling exceeds the driving force for the eutectic reaction. Instead of the growth of primary crystals, the co-growth of eutectic phases is favored by mutual atom exchange between two eutectic phases. Solid-state diffusion during the rapid cooling is suppressed, and thus the precipitation of ordered Ni_3_Si is hindered, resulting in the formation of supersaturated (Ni).

Figure 5e,h show the microstructure and morphology of the Ni19Cr12Si and Ni21Cr11Si. It becomes apparent that the thermal heat extraction plays a defined roll in the appearance of the resulting microstructure, thus it shows that different solid growth mechanisms can be accessed which significantly change the morphology and present phases. The predicted equilibrium phases differ significantly from the observed rapid solidification samples. Based on the reaction scheme for the Ni-Cr-Si system reported by Schuster and Du [36], a likely phase formation pathway for observed microstructure is proposed. Here, (Ni) and an intermediate phase, τ_1,_ form first from the melt (L → (Ni) + τ_1_) followed by a ternary peritectic reaction (L + (Ni) + τ_1_ → π-Cr_3_Ni_5_Si_2_) [13,36]. The peritectic reaction should start at the grain boundaries between (Ni) and τ_1_ phase, and progress outwards, which explains the enveloped structure of π-Cr_3_Ni_5_Si_2_ grains surrounded by (Ni) phase in Figure 5c,d. The solidification ends with another ternary peritectic transition (L + τ_1_ → π-Cr_3_Ni_5_Si_2_ + γ-Ni_31_Si_12_) which consumes the remaining τ_1_ phase.

At a high cooling rate of 11,000 K s^−1^, τ-Cr_6_Ni_16_Si_7_ phase appears in the microstructure rather than γ-Ni_31_Si_12_ and π-Cr_3_Ni_5_Si_2_. It is very likely that γ-Ni_31_Si_12_ and π-Cr_3_Ni_5_Si_2_ phases are the product of decomposition of τ-Cr_6_Ni_16_Si_7_ phase, which was prohibited by rapid cooling. However, it is ambiguous whether τ-Cr_6_Ni_16_Si_7_ phase or τ_1_ phase is in phase equilibrium since the stability of τ-Cr_6_Ni_16_Si_7_ phase in Ni-Cr-Si system is not established [15,36]. Nevertheless, the phase transformation pathway was severely altered by increasing the cooling rates indicated by the presence of metastable high-temperature ternary phases [13] in the terminal microstructure see Figure 5f,h.

The results from the in situ micro-diffraction measurements confirm that the terminal microstructures shown in Figure 5 are inherited from the rapid solidification microstructure at high temperatures. The presence of τ-Cr_6_Ni_16_Si_7_ at room temperature is assumed to stem from an unimpeded phase transformation in the solid-state. The summary of observed solidification and solid-state transformation pathways of each alloy is summarized in Table 3.

Cooling rates do not only affect the phase transformation but also have effects on the morphology of the microstructure. For the Ni14Cr12Si alloy at high cooling rates, contrary to the well-established equilibrium case, diffusion-assisted coarsening is constrained. Hence, as the cooling rates increases, microstructural refinement is observed in all alloys, for instance, smaller primary phase, smaller lamellae, and smaller colony size. Besides, high cooling rates that lead to anomalous eutectic structure due to the decoupled growth of different phases. The regular eutectic to anomalous eutectic transition happens in the system where a disordered phase coexists with an ordered faceted intermetallic compound as its terminal phases [37]. The growth rate (V_growth_) depends on undercooling ($x_{e}^{L/s}-x_{0}^{L}$) and the growth constant (μ), i.e., $V_{growth}=\mu (x_{e}^{L/s}-x_{0}^{L})$, where $x_{e}^{L/s}$ is the solute concentration in the liquid ahead of solidification front and $x_{0}^{L}$ is the initial concentration in the melt.

Compared with intermetallic compounds, a disordered solid solution has a ~2 orders of magnitude higher kinetic coefficient. This coefficient is closely associated with the complexity of the unit cell and the interface attachment kinetics during growth [35]. For the intermetallic phase, different atoms occupy defined positions in each sublattice, so the atom attachment is more restricted, and the growth kinetics are more sluggish. As a result, the atom mobility at the interface of intermetallic compounds is more sluggish compared to that of the disordered phase [38]. The leading phase will grow freely into the undercooled melt and results in a decoupled growth. Anomalous eutectic microstructure with a mosaic-like morphology is common in the cross section of the sample since the dendritic growth of the leading phase is unfavorable by the chemical composition limitation of a eutectic alloy [39]. When a negative temperature gradient is reached during further growth of the colony, decoupled growth is terminated and coupled growth with a planar front takes place again to yield lamellae. This accounts for the observed morphology where lamellae alternate with the anomalous eutectic microstructure as displayed in the inset in Figure 5h, i.e., lamellae growth on the plate.

The addition of Cr and Si promotes the formation of silicides, i.e., π-Cr_3_Ni_5_Si_2_ phase at the low cooling rate and τ-Cr_6_Ni_16_Si_7_ phase at the high cooling rate, respectively. For alloys with compositions close to the eutectic valley (Figure 1), full eutectic structure without primary crystallization formed at high cooling rates and effectively refined the microstructure. At the high cooling rate, a slight composition modification can lead to a completely different microstructure as shown in Figure 5f,h. The change in chemical composition results in the same solidus temperature but different liquidus temperature and consequent difference in solidification interval. The solidification interval is 30 K for Ni19Cr12Si while 15.9 K for Ni21Cr11Si. This determines the level of impurities and constitutional undercooling in front of the advancing interface and has a big impact on interface stability. A large solidification interval usually leads to the occurrence of an unstable solidification front [40].

## 5. Conclusions

Investigating new alloys for laser deposition technologies such as AM proves difficult and costly. This study shows a unique way to investigate non-equilibrium microstructures that occur during the rapid melting and solidification of metallic materials. Four Ni-Cr-Si alloys were selected via thermodynamic simulations and investigated using the presented liquid droplet rapid solidification setup. The solidification pathways were identified using the in situ synchrotron microbeam X-ray diffraction setup. The observed non-equilibrium microstructures were characterized carefully post mortem. Furthermore, a widely used Ni-Cr-Si alloy for laser cladding was investigated and compared to liquid droplet samples.

The effect of cooling rates and chemical composition on the microstructural formation and underlying phase transformation mechanisms in Ni-Cr-Si alloys were investigated. The main conclusions are as follows:

1. A small batch investigation using the liquid droplet technique shows a promising route to investigate new alloys for AM technologies.
2. It has been successfully demonstrated that the microstructure of the laser coating can be estimated by the arc melted rapid solidification experiments. Therefore, it allows pre-selection of alloys suitable for various laser beam-based processes with differences in cooling rates. Here, a broad spectrum of cooling rates was presented e.g., between 1600 K s^−1^ to 111,000 K s^−1^.
3. During rapid cooling, the microstructure mainly consists of anomalous eutectic structures. The cooperative growth mechanism is disrupted by decoupled growth of the present phases which leads to a change in lamellar width and growth of one phase into the melt.
4. The phase transformation pathways occurring during rapid solidification were investigated for high cooling rates in the range of 10^3^–10^4^ K s^−1^ for Ni19Cr12Si and Ni21Cr11Si alloys. Here an increase in cooling rate results in the presence of the metastable τ-Cr_6_Ni_16_Si_7_ phase rather than π-Cr_3_Ni_5_Si_2_ + γ-Ni_31_Si_12_.

## Figures and Tables

**Table 1:** Solidus/liquidus temperature and latent heat of fusion of Ni-Cr-Si alloys as measured by DSC.

Alloys	T_S_ (K)	T_L_ (K)	Heat of Fusion (J g^−1^)
Ni7Cr11Si	1411	1431	156.8
Ni14Cr12Si	1364	1422	161.0
Ni19Cr12Si	1364	1394	176.9
Ni21Cr11Si	1365	1380	161.9

**Table 2:** Phases observed in the alloys after solidification at different cooling rates and phases predicted according to Scheil simulations.

Alloys	r = 2 mm (1600 K s^−1^)	r = 0.5 mm (11,000 K s^−1^)	Scheil Simulation
Ni7Cr11Si	(Ni) + γ-Ni_31_Si_12_		(Ni) + γ-Ni_31_Si_12_
Ni14Cr12Si	(Ni) + γ-Ni_31_Si_12_		(Ni) + γ-Ni_31_Si_12_ + τ_1_-Cr_2_Ni_2_Si_1_
Ni19Cr12Si	(Ni) + γ-Ni_31_Si_12_ + π-Cr_3_Ni_5_Si_2_	(Ni) + τ-Cr_6_Ni_16_Si_7_	
Ni21Cr11Si			

**Table 3:** Observed phase transformation sequences in the smallest spheres (r = 0.5 mm, 11,000 K s^-1^) during rapid solidification and cooling.

Alloys	Observed Phase Sequence	Predicted Phase Sequence (Scheil)
Ni7Cr11Si	L → (Ni) + γ-Ni_31_Si_12_	L → (Ni) → (Ni) + γ-Ni_31_Si_12_
Ni14Cr12Si	L → γ-Ni_31_Si_12_ (P) → γ-Ni_31_Si_12_ (P) + (Ni) + γ-Ni_31_Si_12_ (U)	L → γ-Ni_31_Si_12_ → γ-Ni_31_Si_12_ + (Ni) → γ-Ni_31_Si_12_ (P) + (Ni) + τ_1_-Cr_2_Ni_2_Si_1_
Ni19Cr12Si	L → (Ni) + τ-Cr_6_Ni_16_Si_7_	L → γ-Ni_31_Si_12_ → γ-Ni_31_Si_12_ + (Ni) → γ-Ni_31_Si_12_ + (Ni) + τ_1_-Cr_2_Ni_2_Si_1_
Ni21Cr11Si	L → (Ni) + τ-Cr_6_Ni_16_Si_7_	L → (Ni) → (Ni) + γ-Ni_31_Si_12_ → γ-Ni_31_Si_12_ + (Ni) + τ_1_-Cr_2_Ni_2_Si_1_

Please note that P stands for primary phase, U for phases from binary eutectic reactions.

## Supplementary Materials

The following are available online at https://www.mdpi.com/1996-1944/13/9/2192/s1, Table S1: List of crystallographic parameters of phases referred to in the present work, Table S2: Table of relevant invariant reactions, Figure S1: 2D pattern of amorphous ring of hot melt at t = 4005 ms (left image) and diffraction spots of solidified crystals (right image) at t = 4029 ms in the Ni14Cr12Si alloy.
