# Supplementary material for: In Situ and Ex Situ Characterization of the Microstructure Formation in Ni-Cr-Si Alloys during Rapid Solidification—Toward Alloy Design for Laser Additive Manufacturing
Source: Materials (Basel). 2020 May 10;13(9):2192. doi: 10.3390/ma13092192 (PMC7362108; doi:10.3390/ma13092192)
Supplement: Supplementary file 1 [file materials-13-02192-s001.pdf]

materials
MDPI

Supplementary Materials

In Situ and Ex Situ Characterization of the Microstructure Formation in Ni-Cr-Si Alloys during Rapid Solidification – toward Alloy Design for Laser Additive Manufacturing

Table S1. List of Crystallographic Parameters of Phases Referred in Present Work [Liu, X., et al.: Experimental Investigation of Phase Equilibria in the Ni-Cr-Si Ternary System. J. Phase Equilibria Diffus. 35, 334–342 (2014); Gupta, K.P.: The Cr-Ni-Si (Chromium-Nickel-Silicon) System. J. Phase Equilibria Diffus. 27, 523–528 (2006).].

Phase	Type	Pearson Symbol	Space Group	*a* [Å]	*b* [Å]	*c* [Å]	*β* [°]
(Ni)	Cu	*cF4*	*Fm-3m*	3.552	–	–	90
β_1-Ni_3Si	$AuCu_3$	*cP4*	*Pm-3m*	3.50	–	–	90
β_3-Ni_3Si	–	*mC16*	–	7.04	6.26	5.08	48.84
γ-$Ni_{31}Si_{12}$	$Ni_{31}Si_{12}$	*hP43*	*P321*	6.67	–	12.28	90
δ-Ni_2Si	Co_2Si	*oP12*	*Pnma*	7.06	4.99	3.72	90
σ-$Cr_{13}Ni_5Si$	σ(Cr,Fe)	*tP30*	*P42/mnm*	8.787	–	4.570	90
π-$Cr_3Ni_5Si_2$	$AlAu_4$	*cP20*	*P213*	6.120	–	–	90
τ-$Cr_6Ni_{16}Si_7$	$Mg_6Cu_{16}Si_7$	*cF116*	*Fm-3m*	11.10	–	–	90

Table S2. Table of relevant invariant reactions [Schuster, J.C., Du, Y.: Experimental investigation and thermodynamic modeling of the Cr-Ni-Si system. Metall. Mater. Trans. A. 31, 1795–1803 (2000)].

Ni-Si	Ni-Cr-Si
e7: L ↔ θ + δ-Ni_2Si, T = 1240 °C	E1: L ↔ Cr_3Si + γ-$Ni_{31}Si_{12}$ + δ-Ni_2Si T = 1138 °C
e9: L ↔ (Ni) + β_3-Ni_3Si, T = 1151 °C	E2: L ↔ (Ni) + π-$Cr_3Ni_5Si_2$ + γ-$Ni_{31}Si_{12}$ T = 1082 °C
p5: L + γ-$Ni_{31}Si_{12}$ ↔ β_3-Ni_3Si, T = 1199 °C	U6: L + Cr_3Si ↔ γ-$Ni_{31}Si_{12}$ + τ_1 T = 1126 °C
-	U8: L + β_2- ↔ (Ni) + γ-$Ni_{31}Si_{12}$ T = 1119 °C
-	U9: L + τ_1 ↔ γ-$Ni_{31}Si_{12}$ + π-$Cr_3Ni_5Si_2$ T = 1088 °C

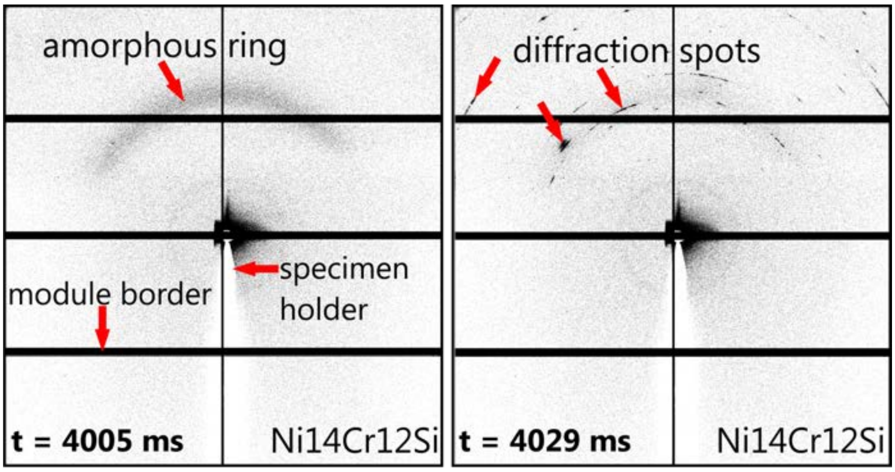

Figure S1. 2D pattern of amorphous ring of hot melt at t = 4005 ms (left image) and diffraction spots of solidified crystals (right image) at t = 4029 ms in the Ni14Cr12Si alloy.
